# Supplementary figures and images for: Clinical presentation and outcomes of the first patients with COVID-19 in Argentina: Results of 207079 cases from a national database
Source: PLoS One. 2021 Feb 11;16(2):e0246793. doi: 10.1371/journal.pone.0246793 (PMC7877635; doi:10.1371/journal.pone.0246793)

**S1 Appendix. COVID 19 suspect o confirm case report. Argentina.**
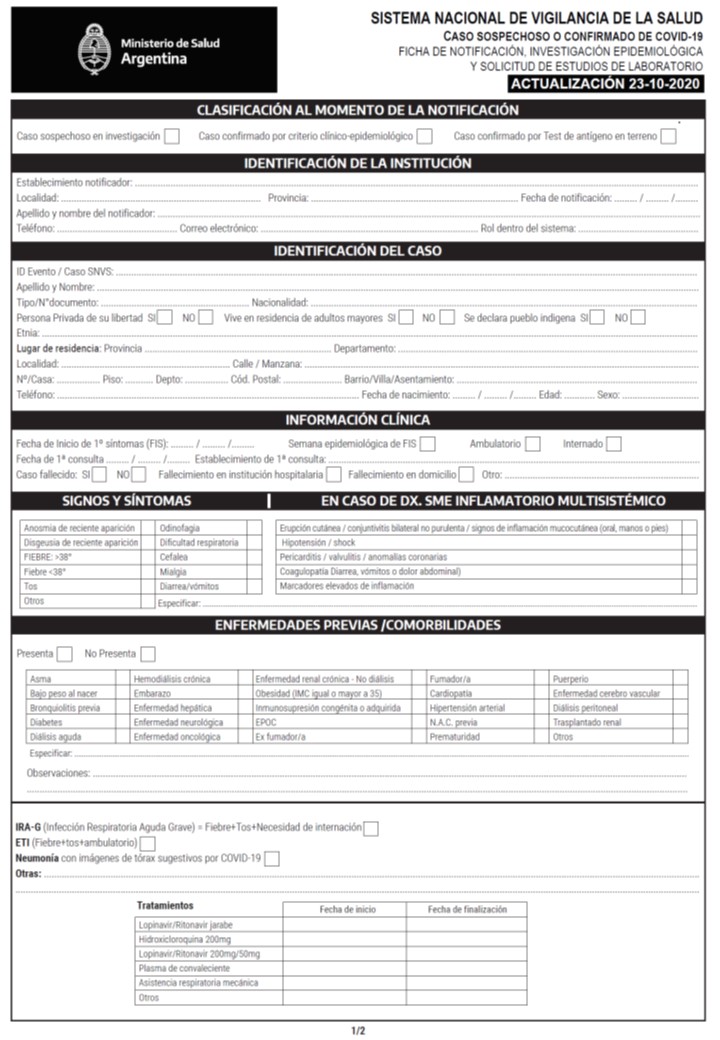


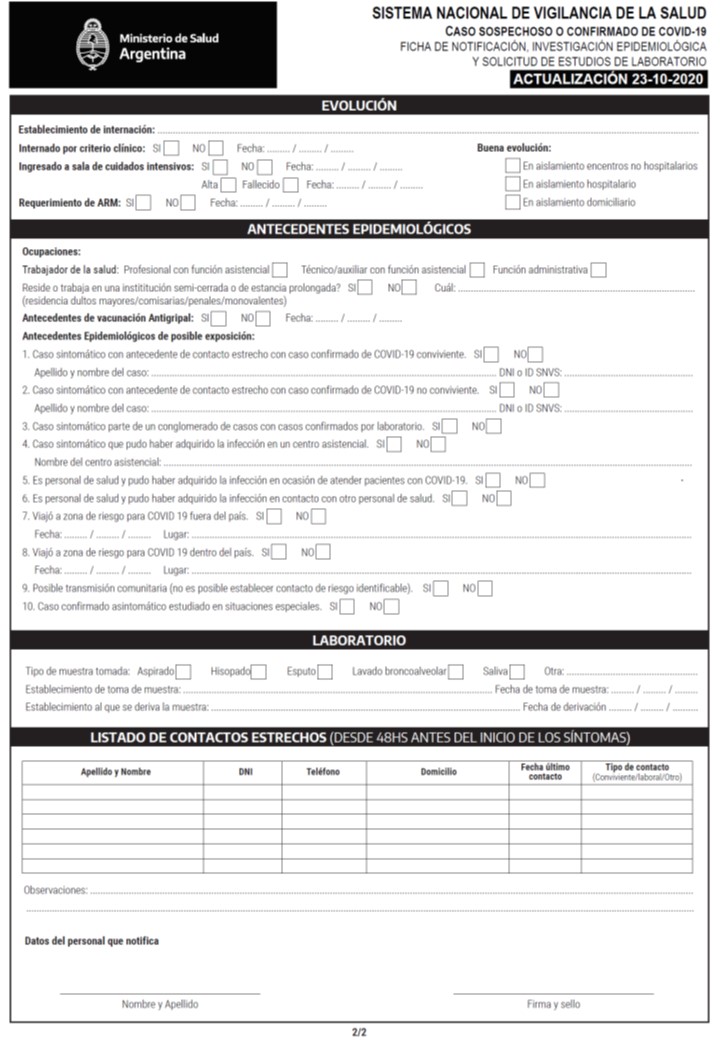

Supplement: S1 Appendix — Argentina. (DOCX) [file pone.0246793.s001.docx]
