# Supplementary material for: Clinical presentation and outcomes of the first patients with COVID-19 in Argentina: Results of 207079 cases from a national database
Source: PLoS One. 2021 Feb 11;16(2):e0246793. doi: 10.1371/journal.pone.0246793 (PMC7877635; doi:10.1371/journal.pone.0246793)
Supplement: Attachment — Submitted filename: Response to Reviewers - COVID paper sisa.docx [file pone.0246793.s002.docx]

Response to Reviewers

Francesco Di Gennaro
Academic Editor
PLOS ONE

Dear Academic Editor,

Thank you for giving careful consideration to our paper “Clinical presentation and outcomes of the first patients with COVID-19 in Argentina: results of 207079 cases from a national database” (PONE-D-20-38991)

We included our action to any question or comments send for you or reviewers in this updated letter. For best organization, the response is ordered by reviewers.

**Academics Editor**

1.) Please ensure that your manuscript meets PLOS ONE's style requirements, including those for file naming. The PLOS ONE style templates can be found at

***All manuscript was controlled to adequate to PLOS ONE style requeriments.***

2.) Thank you for stating the following in the Financial Disclosure section: 'The author(s) received no specific funding for this work.'

***Included***

a.) Please provide an amended Funding Statement declaring this commercial affiliation, as well as a statement regarding the Role of Funders in your study.
b.) Please also provide an updated Competing Interests Statement declaring this commercial affiliation along with any other relevant declarations relating to employment, consultancy, patents, products in development, or marketed products, etc.

***“Centro Diagnóstico San Jorge. Puerto Madryn” did not play a role in the study design, data collection and analysis, decision to publish, or preparation of the manuscript and only provided financial support in the form of one of authors' salaries.***

***The author contributions roles were reviewed and accurately indicated.***

***The funder provided support in the form of salaries for authors [DS, SA, JCB, HF, DG, DPCh], but did not have any additional role in the study design, data collection and analysis, decision to publish, or preparation of the manuscript. The specific roles of these authors are articulated in the ‘author contributions’ section.***

***Regarding commercial affiliation “This does not alter our adherence to PLOS ONE policies on sharing data and materials”***

3.) We note that you have indicated that data from this study are available upon request. PLOS only allows data to be available upon request if there are legal or ethical restrictions on sharing data publicly.

a.) If there are ethical or legal restrictions on sharing a de-identified data set, please explain them in detail (e.g., data contain potentially identifying or sensitive patient information) and who has imposed them (e.g., an ethics committee). Please also provide contact information for a data access committee, ethics committee, or other institutional body to which data requests may be sent.

***There are legal restrictions on sharing the dataset. The dataset is an official database of COVID 19 pandemic in Argentina and authorization about use, analysis and public diffusion of this date, can only be approved by the Argentinian government.***

***The contact information of a data access is the “Dirección Nacional de Epidemiología e Información Estratégica” – email:*** [***nuevosnvs2@gmail.com***](mailto:nuevosnvs2@gmail.com)***. Information Access might be requested from the Instituto Nacional de Enfermedades Respiratorias Emilio Coni – email:*** [***direccionconi@gmail.com***](mailto:direccionconi@gmail.com)***.***

4.) Please include captions for your Supporting Information files at the end of your manuscript, and update any in-text citations to match accordingly.

***Included***

**Reviewer 1**

Reviewer #1: Authors wrote a very strong article. The number is incredible. Well done. Only some minor suggestions

1. Introduction: add data on covid burden at the day of resubmission

***Included in reviewed manuscript:***

***“On January 10^th^ in 2021, the Ministry of Health reports: 1.714.409 confirmed cases, with 1.504.330 patients had recovered and 44.417 died.***

2. Methods and results no comment.

***No comment***

3. table and figure are clear.

***No comment***

4. Disucssion. Compare your data with other paper with high number of patients (see and cite COvid-19 RISk and Treatments (CORIST) collaboration. Common cardiovascular risk factors and in-hospital mortality in 3,894 patients with COVID-19: survival analysis and machine learning-based findings from the multicentre Italian CORIST Study. Nutr Metab Cardiovasc Dis. 2020 Oct 30;30(11):1899-1913. doi: 10.1016/j.numecd.2020.07.031. Epub 2020 Jul 31. PMID: 32912793.

Copy ; doi: 10.1016/j.ejim.2020.08.019. Epub 2020 Aug 25. PMID: 32859477; PMCID: PMC7446618. and doi: 10.1016/j.vph.2020.106805. Epub 2020 Sep 28. PMID: 32992048; PMCID: PMC7521934.)

***Thanks for the suggestion. Our results about sign and symptoms and comorbidities were compared with many other studies, and their conclusion were included in Discussion section.***

***However, we appreciate recommendation and include CORIST study in comorbidities risk factor in Discussion and include citation in references (reference 40)***

Add also information on publich health approuch in Argentina to contaoin covid spread.

***Included in reviewed manuscript:***

***“To contain the COVID-19 spread, the government implemented a national lockdown as of March 20th, 2020, with various levels of implementation across the country, and is still ongoing at the time of submission.***

***On July 31st, 2020, the Ministry of Health released a report stating the reinforcement of the health system by increasing the number of ICUs beds by 40%, including professionally trained staff and critical care support infrastructure. Twelve new modular hospitals were opened in the geographic areas were most COVID-19 cases seemed to be concentrated.”***

**Reviewer 2**

Reviewer #2: This manuscript has described the clinical symptoms of COVID-19 patients at baseline and the clinical characteristics of patients admitted to the ICU. I think the author has used sufficient data and appropriate statistical analysis in making conclusions. The writing is also in standard English and easy to understand. What is interesting and in my opinion is also important in epidemiology, the author also included data on the number of health workers affected by covid19 (paragraphs 242-244) but unfortunately there is no further data on the type of occupation in the patient as a whole. Smoking is also an important data added value in this manuscript because as we all know the number of smokers in developing countries is very high.

***Unfortunately, we are unable to provide further data on patient’s occupation, other than the health workers. We apologize for this limitation.***

***Smoking was included precisely in light of the insightful reviewer’s comment. In Argentina, the proportion of smokers is high. However, we found no correlation between smoking and severity of COVID-19.***
